# Urbanization, environment and pharmaceuticals: advancing comparative physiology, pharmacology and toxicology

**DOI:** 10.1093/conphys/cox079

**Published:** 2018-01-17

**Authors:** Bryan W Brooks

**Affiliations:** Department of Environmental Science, Institute of Biomedical Studies, Center for Reservoir and Aquatic Systems Research, Baylor University, Waco, TX, USA

**Keywords:** Urbanization, one health, coupled health and ecological, hazard and risk assessment, adverse outcome pathways, sustainable development goals

## Abstract

Pharmaceuticals are routinely reported in the environment, which indicates an increasingly urban water cycle and highlights a global megatrend. Physicochemical properties and intrinsic biological activity of medicines routinely differ from conventional organic contaminants; thus, diverging applicability domains often challenge environmental chemistry and toxicology computational tools and biological assays originally developed to address historical chemical stressors. Because pharmacology and toxicology information is more readily available for these contaminants of emerging concern than other chemicals in the environment, and many drug targets are conserved across species, leveraging mammalian drug discovery, safety testing and clinical pharmacology information appears useful to define environmental risks and to design less hazardous industrial chemicals. Research is needed to advance biological read across, which promises to reduce uncertainties during chemical assessment aimed at protecting public health and the environment. Whereas such comparative information has been critical to advance an understanding of pharmaceutical hazards and risks in urban ecosystems, studies of medicines with fish and other ecotoxicological models are reciprocally benefiting basic and translational efforts, advancing comparative mechanistic toxicology, and providing robust comparative bridges for integrating conservation and toxicology.

## Teaser

Pharmaceuticals are indicators of a globally urbanizing water cycle. Unique physicochemical properties and biological activities of medicines diverge from traditional environmental contaminants, and thus challenge historical assessment approaches. Studies to understand environmental risks of pharmaceuticals benefit from mammalian pharmacology and toxicology data, and reciprocally advance basic and applied comparative efforts.

## Pharmaceuticals in urban ecosystems

Earth is an increasingly urban planet with population growth projections to ~9.8 billion by 2050 (www.un.org). With more people now living in cities, by 2050 over 70% of the global population will reside in urban areas, reflecting a global megatrend (www.un.org). Such higher densities of human populations inherently results in a concentration of consumption, including consumer chemical products, in cities. Urbanization further brings physical, chemical and biological modification of watersheds and surface waters receiving discharges of reclaimed wastewater or untreated sewage, resulting in instream flows that can be dominated by or even dependent on effluent discharges (Brooks *et al.*, 2006). These urban ecosystems are now recognized as important management units in the face of climate change (Luthy *et al.*, 2015) where low dilution of down the drain chemicals present risks to ecosystems and downstream users (Brooks *et al.*, 2006; Ankley *et al.*, 2007; Rice and Westerhoff, 2017). Understanding urban metabolism and designing products, infrastructure and systems to improve resiliency and sustainability of resources, while reducing chemical risks to public health and the environment, represent critically important grand challenges to achieve a number of the United Nations’ Global Goals for Sustainable Development (www.globalgoals.org) and related conservation needs.

Pharmaceutical residues in the environment are indicators of increasingly urban ecosystems. Two decades ago studies of pharmaceuticals in the environment in general, and aquatic ecosystems in particularly, were rare. One of our earliest posters on the subject was presented at a Society of Environmental Toxicology and Chemistry meeting ~18 years ago. During that meeting I recall only one other poster presentation (by Chuck Erikson from the U.S. Food and Drug Administration) on the topic. We were placed together at the very end of the line of poster numbers, on the last day of the meeting. Since this time, numerous investigators have studied pharmaceuticals in the environment. For example, a recent Web of Science search with the terms ‘aquatic’ and ‘pharmaceutical’ yielded just nine manuscripts in 1996, but exponentially grew to 2694 papers by 2006, and then continued to 25 957 articles in 2016. Diverse scientific and engineering, social science and humanities disciplines have engaged this topic with efforts ranging from education programs, the arts, analytical methodologies, environmental fate and exposure pathways, ecological and human health toxicology studies, and interventions ranging from chemical substitutions and drug take back programs to green chemistry and development of innovative engineering technologies. Buoyed by recognition of antimicrobial resistance as a leading global public health threat and recent global key question exercises (Boxall *et al.*, 2012; Rudd *et al.*, 2014), ongoing efforts continue to pursue these subjects, and further aim to develop mechanistic understanding of bioaccumulation dynamics and toxicity pathways to prioritize attributes of chemicals for advanced research, innovation and management (Brooks and Steele, 2018).

### Pharmaceuticals as model substances

When we initiated studies to understand ecological hazards and risks of medicines in urban ecosystems, unique chemical attributes (e.g. most pharmaceucticals are ionizable in aquatic ecosystems) (Valenti *et al.*, 2011) and intentionally designed biological activities of these contaminants of emerging concern presented challenges to employing many historical environmental chemistry modelling and ecotoxicological approaches. Available tools were previously developed for non-ionizable organic contaminants, metals and other traditional aquatic stressors (e.g. ammonia, depressed dissolved oxygen)—markedly different contaminants than pharmaceuticals (Brooks *et al.*, 2003b; Ankley *et al.*, 2007; Brooks *et al.*, 2009a, 2012a, 2012b; Brooks, 2014).

The field of ecotoxicology, essentially aggregated from diverse fields, most notably ecology, physiology and the agricultural and medical disciplines, had historically been considered a largely descriptive exercise by some. In fact, 20 years ago short term (e.g. <96 h) experimental designs with morphometric responses (e.g. survival, growth) to contaminants were the norm, and mechanistic studies were rare. Ecotoxicologists often would look for more mechanistic information from the biomedical literature to formulate testable hypotheses and interpret their research findings. Subsequently, relatively few ecotoxicity mechanisms and pathways (or, adverse outcome pathways (AOP), responses from chemical molecular initiation to adverse outcome for an individual or population) (Ankley *et al.*, 2010) were well understood. Such mechanistic ecotoxicology studies with pharmaceuticals were similarly lacking, and when available, were essentially limited to endocrine active medicines (Arcand-Hoy *et al.*, 1998). Unfortunately, even mechanistic studies with therapeutics targeting endocrine function were almost non-existent at the time (Nimrod and Benson, 1997).

It is important to note that historical ecotoxicology tools were being prescriptively applied (and still are routinely used, with some slow improvements) by regulatory agencies to evaluate chemical contaminants, including pharmaceuticals. Take, for example, the cladoceran models, *Ceriodaphnia dubia* and *Daphnia magna*. Standardized experimental designs allow for robust investigations of contaminant effects on cladoceran survival and reproduction in 7 or 21 day studies, respectively. These invertebrate reproduction responses are quite sensitive to many contaminants, and are thus commonly used to study chemicals prior to introduction to commerce and retrospectively for existing environmental contaminants or evaluations of wastewater effluent discharge and ambient surface water quality. Because concerns had been raised regarding potential influences of mammalian estrogen and androgen agonists on endocrine function of invertebrates, we examined multi-generational *D. magna* responses to the synthetic estrogen agonist 17α-ethinylestradiol (EE2) using biochemical, individual and population level endpoints (Clubbs and Brooks, 2007). Traditional survival, growth and reproduction and biochemical biomarker thresholds were observed at mg/L treatment levels, which are markedly higher than EE2 concentrations in aquatic systems (Clubbs and Brooks, 2007). Yet if aquatic vertebrate responses to EE2 are considered, then adverse reproduction thresholds are observed at 6 orders of magnitude lower, and environmentally relevant, concentrations (Caldwell *et al.*, 2008). These diverging observations are explained by the absence of estrogen receptors in invertebrates, and so EE2 likely exerts toxicity to cladocerans through a non-specific mechanism of action (e.g. narcosis) while specifically acting in vertebrates by agonizing estrogen receptors. Such differential sensitivity was clearly illustrated by fish population declines in a natural lake following treatment with low ng/L levels of EE2 (Kidd *et al.*, 2007). Thus, an understanding of comparative biology is critical when selecting a bioassay model and responses during ecotoxicology studies with pharmaceuticals and other specifically acting chemicals.

Imagine what we could learn through the study of pharmaceuticals in urban ecosystems. This idea permeated our early thoughts and has continued to influence our experimental and theoretical studies. Fortunately, more information exists for pharmaceuticals than other classes of environmental contaminants. Because human therapeutics are extensively tested during drug development (Seiler, 2002), it was immediately clear how useful pre-clinical pharmacology and toxicology information could be to assist ecologically focused efforts. Therapeutics had long been used (e.g. as positive controls) during basic and applied physiological experiments. Similarly, evaluations of pharmaceutical safety had historically been performed with non-human mammals and, more recently, alternative (e.g. fish) models. We recognized the unique opportunity to advance comparative physiology, pharmacological and toxicology efforts as we aimed to understand ecological hazards and risks from pharmaceuticals in urban ecosystems. Due to the evolutionary conservation of numerous drug targets across species (Huggett *et al.*, 2003; Gunnarsson *et al.*, 2008; Verbruggen *et al.*, 2017), we embraced both basic and applied comparative studies. In addition to understanding whether adverse ecological outcomes occur following pharmaceutical exposure, pharmaceuticals could serve as experimental ‘scalpels’ to comparatively probe hundreds of previously understudied toxicity pathways in aquatic and terrestrial organisms. For example, Ankley *et al.* (2009) identified a number of endocrine active chemicals, including several pharmaceuticals, with diverse MOAs to probe potential adverse outcomes.

Of particular relevance to achieving conservation and related sustainable development goals, advancing comparative physiology, pharmacology and toxicology is critical to rationally design industrial chemicals with unintended hazards. In fact, sustainable molecular design is inspired by the fourth principle of green chemistry (Anastas and Warner, 1998). Herein, lessons learned from the design of less hazardous medicines promise to support sustainable molecular design of less hazardous environmental contaminants (Coish *et al.*, 2016, 2018). Our efforts with pharmaceuticals in aquatic ecosystems have thus attempted to advance an understanding of how unique attributes of these contaminants influence physicochemical behaviour and activity in biological systems, rather than simply testing specific substances for generation of descriptive information.

## Pharmaceutical ecotoxicology studies and biological read-across

Of the over 87 000 chemicals in commerce, empirical toxicology data exists for very few environmental contaminants. Costs, animal welfare concerns and an ever-changing chemical space in commerce represent palpable barriers to obtaining robust hazard data in a timely manner. Herein, ‘chemical read-across’, chemical grouping, and intelligent testing strategies are routinely used to fill in data gaps during regulatory risk assessments of chemicals (Barratt, 2003; Van der Jagt *et al.*, 2004; van Leeuwen *et al.*, 2007). For example, Bradbury *et al.* (2004) considered chemical read-across methods, which essentially presume that similarities among molecular structures result in similar physicochemical properties, biological activity and toxicity, pragmatic for predicting toxicity of untested chemicals, particularly when knowledge of molecular initiating events are understood across chemical classes. Future chemical read-across applications are being improved through next generation in silico computational toxicology models, which are expanding earlier quantitative structure activity relationship tools (McRobb *et al.*, 2014).

‘Biological read-across’ represents a comparative approach that can be useful to: (i) define pharmacokinetic and toxicokinetic and dynamic differences among species and *in vitro* systems; (ii) improve mechanistic integration in environmental hazard and risk assessment practice; and (iii) select alternatives and design less hazardous chemicals. As noted above, a wealth of information exists, though not completely in the public domain, for pharmaceuticals compared to other environmental contaminants (Seiler, 2002). Building from earlier efforts (Brooks *et al.*, 2003a; Huggett *et al.*, 2003; Foran *et al.*, 2004), we (Brain *et al.*, 2008; Brooks *et al.*, 2009b; Berninger and Brooks, 2010) and others (Owen *et al.*, 2007; Gunnarsson et al., 2008; Winter *et al.*, 2009; Fick *et al.*, 2010; Rand-Weaver *et al.*, 2013) have further explored biological read-across to define hazards and risks of pharmaceuticals in urban ecosystems. Herein, contributions by Duane Huggett, Joakim Larsson and Stewart Owen are particularly important. Subsequently, an expert workshop with scientists from academic, government and industry recently identified 20 important questions that, if answered, would support an understanding of environmental risks posed by pharmaceuticals and personal care products (PPCP) (Boxall *et al.*, 2012). Simply stated, such priority questions from the scientific community have effectively proposed a global research agenda for the field, having benefited from similar efforts in conservation biology (Sutherland *et al.*, 2009). Several research questions were specifically related to biological read across. For example, answering the questions, ‘How can pharmaceutical pre-clinical and clinical information be used to assess the potential for adverse environmental impacts of pharmaceuticals?’ and ‘What can be learned about the evolutionary conservation of PPCP targets across species and life stages in the context of potential adverse outcomes and effects?’ are considered key for identifying substances of environmental concern.

Though descriptive bioassay tools continue to be employed during regulatory assessment of chemical contaminants, ecotoxicology has matured to a mechanistic science, largely benefiting from advances in molecular genetics, biochemistry, physiology and computational chemistry. When ecological studies of pharmaceuticals started to appear, descriptive bioassays predominated while some general biomarker studies slowly started to appear in the literature. At this time, the use (and misuse) of biomarkers in retrospective risk assessment was much more common than prospective applications, reflecting an earlier bifurcation between biomarkers of exposure and biomarkers of effect (NRC 1987; Stegeman *et al.*, 1992). Based on lessons learned from studies of endocrine active chemicals, including human and veterinary medicines, Ankley *et al.* (2007) provided some guidance for selection of biomarkers within an ecological risk assessment framework. We (Ankley *et al.*, 2007) further proposed selecting in vivo ecotoxicology models for sublethal chronic studies based on evolutionary conservation of pharmacological targets, pathways and physiological functions across species. Ankley *et al.* (2010) then advanced these ideas and earlier concepts (Bradbury *et al.*, 2004) to propose the AOP conceptual framework for environmental risk assessment. An AOP considers intrinsic chemical properties influencing molecular initiation events (anchor 1 responses), defined when a contaminant interacts with a specific biological target (e.g. receptor, enzyme), and then the subsequent cascading responses along increasing scales of biological organization that result in one or more adverse outcomes at the individual and population levels (anchor 2 responses) of a species (Ankley *et al.*, 2010). Adverse outcomes at these levels of biological organization represent important ecological protection goals for threatened and endangered species and other organisms, respectively. Herein, robust AOPs can support development of hypotheses, design of mechanistic experiments, and an understanding of pathway responses to chemical perturbations. Brausch *et al.* (2012) subsequently suggested that future ecotoxicology studies with pharmaceuticals should employ AOPs.

Several efforts have specifically aimed to test a biological read-across hypothesis using an AOP framework. For example, we (Valenti *et al.*, 2012) observed mechanistic and behavioural anti-anxiety responses in adult male fathead minnows (*Pimephales promelas*) following waterborne exposure to the antidepressant sertraline. In this study, water treatment levels of sertraline were predicted, and then observed, to accumulate in fish plasma at levels exceeding human therapeutic doses (Valenti *et al.*, 2012). When this occurred, serotonin reuptake transporter binding in the brain (an anchor 1 related response), which parallels sertraline activity in mammals, was observed, and shelter seeking behaviour during light conditions (an anchor 2 related response), which mimics elevated plus maze anti-anxiety responses by rodents, were significantly reduced (Valenti *et al.*, 2012). This study thus coupled fish plasma modelling (Huggett *et al.*, 2003) within an AOP framework to extend mammalian observations to fish. In addition to fish behavioral studies with fluoxetine by Weinberger and Klaper (2014), Margiotta-Casaluci *et al.* (2014) reported similar fish behavioural responses to the antidepressant fluoxetine, and appreciably advanced fish plasma coupling to AOPs (termed quantitative AOPs) using synthetic glucocorticoids as examples (Margiotta-Casaluci *et al.*, 2016). These efforts highlight the promise of biological read-across to leverage available mammalian safety data to support ecotoxicological studies of pharmaceuticals. Such observations are instructive because these AOP-informed adverse effect thresholds, much like the EE2 example presented above, are quite lower than those reported for fluoxetine and sertraline using descriptive bioassays employed during routine regulatory assessment of pharmaceutical hazards and risks (Brooks *et al.*, 2003b; Stanley *et al.*, 2007; Valenti *et al.*, 2009).

**Figure 1:**
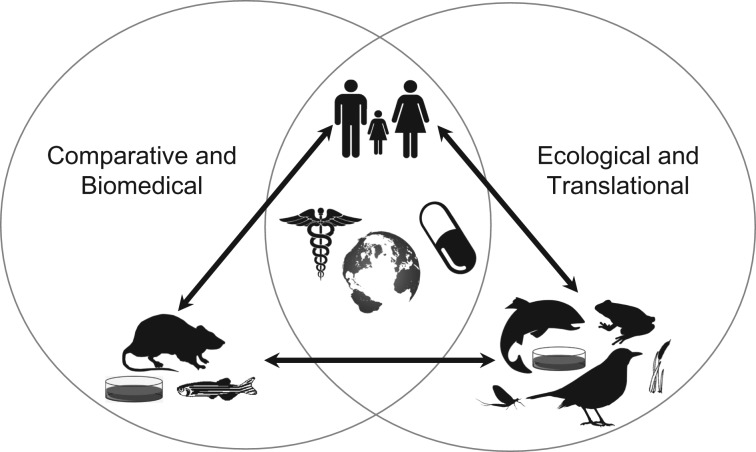
Comparative physiology, pharmacology and toxicology studies of pharmaceuticals in the environment are providing robust bridges that reciprocally benefit basic and translational sciences.

In parallel with efforts to understand ecological consequences of pharmaceuticals in urban systems, a marked increase in the use of fish models for biomedical research has occurred, including studies of chemical carcinogenicity and other toxicology questions (Fig. 1) (Hinton *et al.*, 2009). Fish models are employed during early phases of drug development and safety evaluations due to animal welfare concerns, rapid rates of reproduction, and lower costs of care, husbandry and testing (Powers, 1989). For example, zebrafish are increasingly used in neuropharmacology, where behavioural phenotypes associated with various moods, psychological disorders and social preferences have been reported following exposure to neuroactive chemicals, including pharmaceuticals and pesticides (Stewart *et al.*, 2015; Kalueff *et al.*, 2016). During these biomedical-focused studies, anxiety related wall hugging behaviour and photomotor responses of fish are known to be differentially altered by various therapeutics (Steenbergen *et al.*, 2011; Schnörr *et al.*, 2012; Maximino *et al.*, 2014). Such behavioural endpoints can be used to quickly identify potential molecular initiation events leading to physiological changes observed in an individual (Kokel *et al.*, 2010). In fact, recent advances in digital tracking technology and computational methods are allowing for differential behavioural responses to contaminants and drugs to be rapidly determined in fish embryos and larva (Noyes *et al.*, 2015; Maximino *et al.*, 2014). Coupling underlying mechanisms with such behavioural response profiles is being used to examine drug safety, and reciprocally appears useful to attempt to translate chemical mechanism of action specific behavioural responses of larval zebrafish to other laboratory fish models and life stages (Fig. 1) (Parker, 2016; Kristofco *et al.*, 2016; Steele *et al.*, accepted).

## Future perspectives

Though the United Nations has identified 17 Sustainable Development Goals, we will not realize such noble goals related to conservation if we do not advance sustainable environmental quality. Yet global sustainable environmental quality cannot be achieved without integration of conservation and environmental toxicology and ecotoxicology. In fact, the need for integrative conservation biology and toxicology has been long recognized (Hansen and Johnson, 1999), was identified during development of the scope of ‘Conservation Physiology’ (Cooke *et al.*, 2013), and can be facilitated by behavioural ecology (Peterson *et al.*, 2017). Long held notions by mainstream ecology that ecotoxicology is a simple descriptive enterprise are slowly changing, as toxicology has matured to an increasingly mechanistic science. However, ecotoxicology continues to bifurcate between mechanistic molecular understanding and studies of ecological thresholds to diverse stressors in the field. Herein, it will be critical to ensure conservation efforts are informed by robust mechanistic information, if environmental management goals are to be realized, particularly in rapidly urbanizing ecosystems. Comparative physiology, pharmacology and toxicology studies of pharmaceuticals and the environment are providing robust bridges among mechanistic biomedical, ecological and broader translational sciences (Fig. 1).

Despite a number of promising developments, including quantitative behavioural technologies and AOPs for diverse molecular initiation events, it is important to note there are numerous data gaps, modelling assumptions, and uncertainties associated with the existing state of comparative pharmacology and biological read-across (Brooks, 2014). For example, some of the above examples highlight mechanistic linkages to behavioural perturbations, yet standardized experimental designs for regulatory evaluations of environmental contaminant effects on behaviour are limited. Robust experimental behavioural methods need to be developed for inclusion in regulatory environmental assessments if identification of thresholds of ecologically important adverse outcomes are desired. Future research is also needed to advance integration of comparative toxicity pathways associated with diverse pharmaceutical molecular initiation events. *In vitro* and embryonic fish models represent increasingly used high throughput testing strategies; mechanistically linking responses in these models to adverse outcomes and environmental management goals (e.g. biodiversity, ecosystem services) used in hazard and risk assessment is necessary. Herein, underapp/reciated responses such as disruption of circadian rhythms warrant consideration (Melvin, 2017).

Contemporary studies in this area often include freshwater laboratory fish models, which are commonly used for regulatory or biomedical purposes. Within just one fish model, age specific behavioural baseline differences and toxicological responses are observed in zebrafish larva that are slightly older than those early developmental windows employed for standard regulatory studies (Kristofco *et al.*, 2015, 2016), apparently resulting (at least in part) from increased accumulation and toxicokinetic and toxicodynamic differences with age (Kristofco *et al.*, 2018). Further, between just the two most common laboratory fish larval models, zebrafish and fathead minnows, mechanistic (Corrales *et al.*, 2017) and photomotor behavioural (Steele *et al.*, accepted) responses differ for diverse contaminants, including neurologically active pharmaceuticals and pesticides. Whether such behavioural observations in these common fish models translate to other less commonly studied fish species (Saaristo *et al.*, 2017; Martin *et al.*, 2017) or wild fish responses to neuroactive and other therapeutics or stressors represent important research needs. For example, recent studies have extended laboratory studies with a benzodiazepine (Brodin *et al.*, 2013) to use telemetry to observe fish behaviour in the field following pharmaceutical treatment (Klaminder *et al.*, 2016; Hellström *et al.*, 2016a,b). Behavioural studies with invertebrates and estuarine and marine species are much less common (Fong and Ford, 2014; Pyle and Ford, 2016), yet human populations routinely reside on or immediately upstream from coastlines. Clearly, comparative research is needed for behaviourally modifying pharmaceuticals and other classes of contaminants.

Comparative information has been critical to advance environmental investigations of medicines, yet these studies of pharmaceuticals in aquatic models are also reciprocally benefit basic and translational efforts of relevance to the biomedical and ecological sciences (Fig. 1). Such efforts will become more important across scales as studies of environmental stressors continue to seemingly bifurcate between mechanistic efforts in the laboratory and investigations of ecological thresholds and adverse health outcomes in the field. Further, global megatrends associated with urbanization and the food-energy-water nexus will only become more pronounced, and thus require integrated environment and health approaches. As noted above, some key questions have emerged to understand risks of pharmaceuticals in urban ecosystems (Boxall *et al.*, 2012; Rudd *et al.*, 2014). This horizon scanning approach (Brooks *et al.*, 2013) has been extended to identify diverse research needs of relevance to specific geographic regions (Furley *et al.*, 2018). Answering these questions will of course be non-trivial; however, doing so promises an ushering of step changes in comparative pharmacology and toxicology to predictively diagnose and reduce hazards and risks of pharmaceuticals and other contaminants in urban ecosystems. Imagine what we will learn.
